# Circadian biomarkers in patients with bipolar disorder: promising putative predictors of lithium response

**DOI:** 10.1186/2194-7511-2-5

**Published:** 2014-04-09

**Authors:** Pierre Alexis Geoffroy, Bruno Etain, Sarah Sportiche, Frank Bellivier

**Affiliations:** Inserm, U1144, Paris, F-75006 France; AP-HP, GH Saint-Louis - Lariboisière - Fernand Widal, Pôle Neurosciences, Paris CEDEX, 10 75475 France; Université Paris Diderot, UMR-S 1144, Paris, F-75013 France; Fondation FondaMental, Créteil, 94000 France; AP-HP, Hôpital H. Mondor - A. Chenevier, Pôle de Psychiatrie, Créteil, 94000 France; INSERM, U955, Psychiatrie génétique, Créteil, 94000 France

**Keywords:** Lithium salts, Lithium carbonate, Lithium acetate, Circadian rhythms, Sleep, Chronobiology, Circadian genes, Recurrences, Remission, Mood stabilizers

## Abstract

Bipolar disorder (BD) is a common, severe mental disorder with a high recurrence rate. Lithium (Li) is the cornerstone of BD treatments to reduce recurrence, suicide, and mortality risks. However, only 30% of patients treated with Li achieve complete remission, and few markers of the response to treatment have yet been identified for application in routine practice. Circadian biomarkers may be relevant predictors of individual responses to Li because (1) Li has been shown to affect circadian rhythms, (2) disrupted circadian rhythms are a core expression of susceptibility to BD, and (3) circadian abnormalities during euthymia are associated with relapses.

## Correspondence

Bipolar disorder (BD) is a common, severe mental disorder with an onset before the age of 21 years in half the individuals affected (Geoffroy et al. 2013c; Phillips and Kupfer 2013). BD impairs functioning and decreases health-related quality of life. BD is the fourth most important contributor to the global disease burden among mental, neurological, and substance use disorders (Scientific Advisory Board and the Executive Committee of the Grand Challenges on Global Mental Health et al. 2011). The severity and poor prognosis of BD reflect the high rate of recurrence, with a mean recurrence rate of 60% within 2 years of an index episode, despite medications (Geddes and Miklowitz 2013).

Lithium (Li) is the first-line treatment of BD and the cornerstone of treatment for preventing relapses and recurrences of any episodes of either polarity (Grunze et al. 2009; Yatham et al. 2009; Goodwin 2009; Grunze et al. 2010). Furthermore, it is the only treatment shown to decrease suicide risk effectively (Yerevanian and Choi 2013). Li has also been shown to decrease the risk of non-suicide mortality in patients with BD (Müller-Oerlinghausen et al. 1992). However, naturalistic studies have shown that about 40% of the BD patients treated with Li show no improvement (non-responders, NRs), about 30% are partial responders (PRs), and only 30% are excellent responders (ERs), presenting complete remission for two full years (Solomon et al. 1995; Maj et al. 1995; Baldessarini and Tondo 2000; Garnham et al. 2007). In addition, prophylactic efficacy can be evaluated only after at least 2 years of treatment.

Few reliable and reproducible predictors of individual responses to Li have been proposed: history of prophylactic response to Li in first-degree relatives, course of the episode, and complete remission between episodes (Kleindienst et al. 2005). Other clinical markers have been put forward, but their replication has been inconsistent: manic-depressive sequence, age at onset, predominant polarity, polarity of the first episode, BD subtype, atypical features (mainly psychotic symptoms, interepisodic residual symptomatology, and rapid cycling), comorbidities, and temperaments (Garnham et al. 2007; Grof 2010; Schulze et al. 2010; Pfennig et al. 2010; Rybakowski et al. 2013).

Thus, therapeutic response is variable, and it remains difficult for clinicians to identify the patients most likely to respond before lengthy Li trials. These data highlight the need to identify biomarkers predictive of individual responses to Li, to improve care plans and the prognosis of patients with BD. However, no such predictors have yet been identified for application in routine practice (Geoffroy et al. 2014).

Circadian biomarkers are potential markers for predicting the response to Li because Li is thought to help stabilize circadian rhythms in BD and to prevent circadian rhythm desynchronization (Klemfuss 1992). Indeed, Li slightly lengthens the circadian period of behavioral rhythms and delays the phase of behavioral and physiological circadian rhythms (such as sleep-wakefulness and body temperature rhythms) in many species, from healthy and affected humans to rats (Johnsson et al. 1983; Pflug and Engelmann 1987; Klemfuss and Kripke 1995). The hypothesis of a circadian mode of action for Li is of particular interest because patients with BD present circadian abnormalities in all phases of the disorder (McClung 2007; Harvey 2008; Etain et al. 2011; McClung 2013). Indeed, such abnormalities occur during acute phases of depression (insomnia, early awakening, hypersomnia) and mania (decrease in the need for sleep). In addition, circadian rhythm and sleep disturbances often precede recurrences and may serve as predictors of a new mood episode. Some patients suffer from seasonal recurrences of depression or mania: 25% of BD patients for depression and 15% for mania (Geoffroy et al. 2013a). These circadian abnormalities are also observed during stable phases of normal mood (euthymia) and are thus considered to be a ‘trait’ of BD. During euthymia, BD patients are more likely than healthy controls to present an evening chronotype, hypersensitivity to disruptive rhythms (jet lag, post-partum, shift work, night vigils, etc.), sleep/wake pattern abnormalities (sleep stability, sleep latency and duration, waking after sleep onset, sleep quality, diurnal activity, and daytime dysfunction), biochemical abnormalities, including melatonin secretion (sleep hormone) and melatoninergic hypersensitivity to light (Hallam et al. 2006; McClung 2007,2013).

Several independent genetic association studies have implicated circadian or melatonin pathway genes, such as *CLOCK*, *GSK3β*, *NPAS2*, *ARNTL1*, *PER3*, *NR1D1*, and *ASMT* (Milhiet et al. 2011), in susceptibility to BD. Polymorphisms associated with BD may be responsible for circadian rhythm disturbances, as recently observed for a common variant of *ASMT* associated with BD (Etain et al. 2012; Geoffroy et al. 2013b). Circadian rhythm instability, through its contribution to the underlying neurobiology and genetics of BD, appears to be a major candidate endophenotype for studies aiming to identify the factors associated with treatment response in BD (Hasler et al. 2006).

Li acts at the molecular level, by modulating the dynamics of clock gene expression and protein rhythms in the peripheral tissues and suprachiasmatic nuclei (the central pacemaker of circadian rhythms) (Etain et al. 2011). For example, Li is known to affect the expression of two key circadian genes, inhibiting *GSK3β* expression and activating *Clock* transcription. It has also been shown to rescue the manic-like behavior of mice transgenic for *GSK3β* and *Clock* (Prickaerts et al. 2006; Jope and Roh 2006; Roybal et al. 2007). It is also suggested that the neurobiological mechanisms by which Li influences circadian rhythms may involve Rev-Erbα, the protein product of the *NR1D1* gene, which is a phosphorylation target of GSK3. Li inhibits GSK3 that in turn cannot phosphorylate the Rev-Erbα protein which degrades (Can et al. 2014). Furthermore, Li has demonstrated to improve diurnal activity rhythm and periodic activity alterations in transgenic mice with neuron-specific expression of mutant Polg (D181A) - an animal model with generated chronobiological abnormalities (Kato et al. 2007).

Sleep and circadian rhythms are not only relevant candidates for the prediction of Li response. Many clinical guidelines and documents have been developed governing their exploration in routine clinical practice (Morgenthaler et al. 2007a, b; Schutte-Rodin et al. 2008; Adult Obstructive Sleep Apnea Task Force of the American Academy of Sleep Medicine et al. 2009). Sleep logs or diaries, sleep or morningness-eveningness questionnaires, and actigraphy (a watch-like tool containing an accelerometer) are potentially relevant and easy to implement in routine practice. Actigraphy also appears to be useful for evaluating the therapeutic response as an outcome measure (Morgenthaler et al. 2007a).

In conclusion, disrupted circadian rhythms are a core expression of susceptibility to BD, persistent circadian abnormalities during euthymia are associated with relapses, and Li is known to act on circadian rhythms. Thus, circadian biomarkers are promising candidate biomarkers for the study of individual response to Li but cannot be used yet in clinical practice to predict this response. Future researches that specifically address this issue and prospective studies assessing the predictive value of circadian biomarkers are therefore highly expected. Possible circadian studies of the molecular signature of the response to Li may also help to better understand Li's action and to pave the way for more personalized medicine.
